# Advances in Neural Recording and Stimulation Integrated Circuits

**DOI:** 10.3389/fnins.2021.663204

**Published:** 2021-08-06

**Authors:** Juzhe Li, Xu Liu, Wei Mao, Tao Chen, Hao Yu

**Affiliations:** ^1^College of Microelectronics, Beijing University of Technology, Beijing, China; ^2^School of Microelectronics, Southern University of Science and Technology, Shenzhen, China; ^3^Advanced Photonics Institute, Beijing University of Technology, Beijing, China

**Keywords:** electrical stimulation, biomedical, stimulation artifact, neural recording, closed-loop system

## Abstract

In the past few decades, driven by the increasing demands in the biomedical field aiming to cure neurological diseases and improve the quality of daily lives of the patients, researchers began to take advantage of the semiconductor technology to develop miniaturized and power-efficient chips for implantable applications. The emergence of the integrated circuits for neural prosthesis improves the treatment process of epilepsy, hearing loss, retinal damage, and other neurological diseases, which brings benefits to many patients. However, considering the safety and accuracy in the neural prosthesis process, there are many research directions. In the process of chip design, designers need to carefully analyze various parameters, and investigate different design techniques. This article presents the advances in neural recording and stimulation integrated circuits, including (1) a brief introduction of the basics of neural prosthesis circuits and the repair process in the bionic neural link, (2) a systematic introduction of the basic architecture and the latest technology of neural recording and stimulation integrated circuits, (3) a summary of the key issues of neural recording and stimulation integrated circuits, and (4) a discussion about the considerations of neural recording and stimulation circuit architecture selection and a discussion of future trends. The overview would help the designers to understand the latest performances in many aspects and to meet the design requirements better.

## Introduction of Neural Recording and Stimulation Circuits

The neural prosthesis chip for biomedical use includes the neural/muscular stimulators and neural recording circuits. In these circuits, the stimulator has been widely used in biomedical applications for decades, such as cardiac pacemaking, cochlear/retinal prosthesis, and cell activation (Chen et al., 2010; Sooksood et al., 2011; Noorsal et al., 2012; Wagner et al., 2018; Lee and Im, 2019; Lin and Ker, 2020; Yen and Ker, 2020). The neural recording circuit is also involved in these applications to sense the neural signal or assess stimulation efficacy and the tissue status to enable closed-loop control in simultaneous neural recording and stimulation (Yoshida and Horch, 1996; Blum et al., 2007; Rolston et al., 2009, 2010; Venkatraman et al., 2009; Xu et al., 2012; Ando et al., 2016; Ramezani et al., 2018; Lancashire et al., 2019; Carmona et al., 2020). The circuits for simultaneous neural recording and stimulation are used in neural prostheses, such as the bionic neural link for limb function restoration (Xu et al., 2012; Sadeghi Najafabadi et al., 2020; Żebrowska et al., 2020).

**FIGURE 1:**
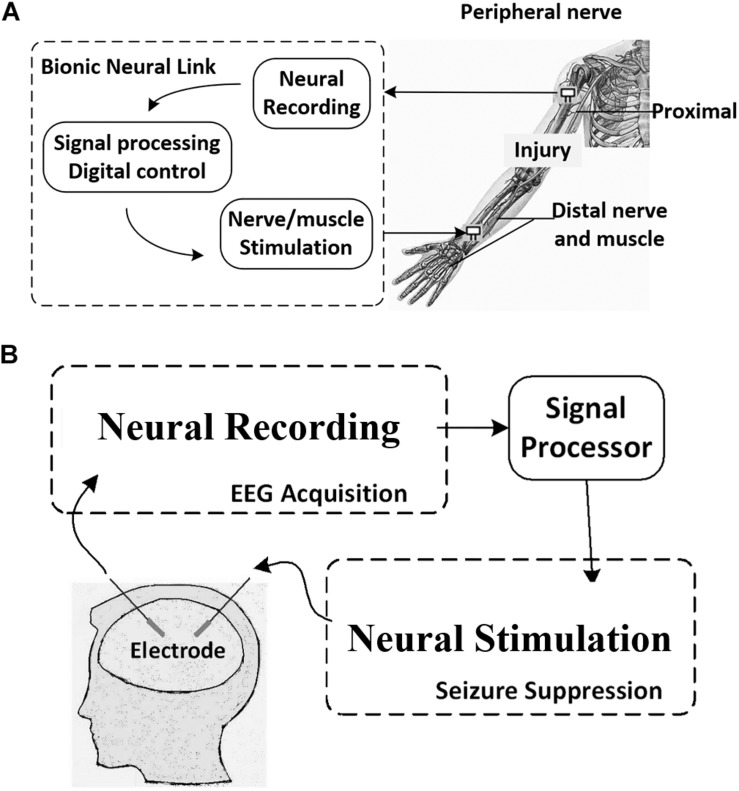
Concept of **(A)** the Bionic Neural Link and **(B)** the epileptic seizure detection and suppression using neural recording and stimulation circuits.

The bionic neural link includes neural recording circuits, stimulation circuits, and action potential (AP) detection circuits (Xu et al., 2017). As shown in Figure 1A, once the AP is detected in the circuit, the bionic neural link bypasses the injury and triggers the stimulator to stimulate the distal nerve/muscle and restore the limb function. The integrated circuit (IC) modules and the working theories will be illustrated in detail in the following sections.

## Advances in Neural Recording and Stimulation Integrated Circuits

### Neural Stimulation

**FIGURE 2:**
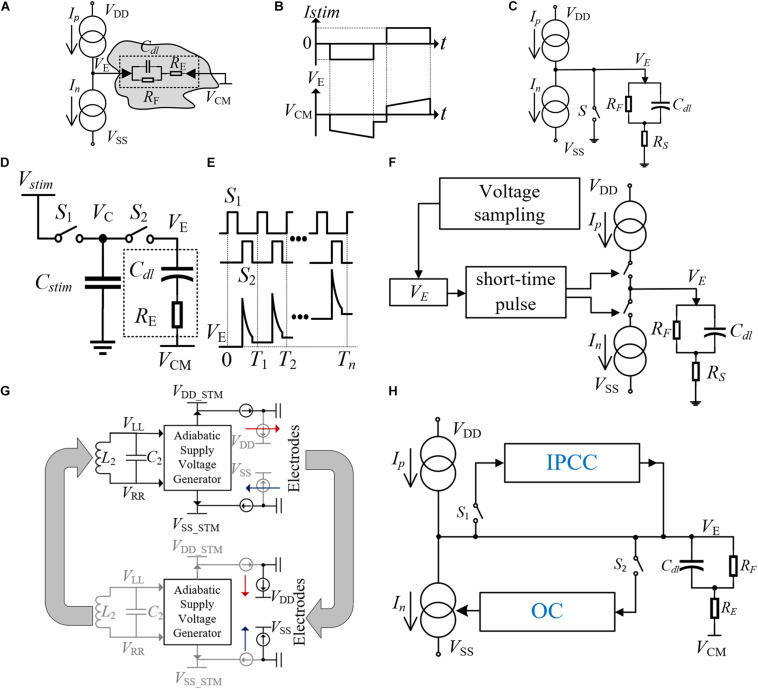
**(A)** Traditional structure of a bidirectional current stimulator. **(B)** The current and voltage waveforms of the bidirectional current stimulator. **(C)** The structure of the stimulator with an electrode short-circuit technique. **(D)** The structure of the high-frequency switched-capacitor stimulator (HFSC). **(E)** The voltage waveform on the electrode with the switch control signals of the stimulator. **(F)** The structure of a stimulator using the short-time pulse insertion technique. **(G)** The structure of the adiabatic stimulator. **(H)** The structure of the stimulator with IPCC and OC.

The essence of electrical stimulation is charge delivery. When the charge accumulation in tissues reaches the threshold, an AP will be produced and muscle contraction will be trigged. The most widely used electrical stimulation method is the current-controlled stimulation (CCS), which benefits from the advantages of controllable charge and high integration. The traditional bidirectional current stimulation scheme is shown in Figure 2A, which consists of two highly matched current sources (*Ip* and *In*), an electrode for stimulating charge transfer (V_E_ is the voltage of electrode), and an electrode for providing a reference voltage V_CM_ (Changhyun and Wise, 1996; Liu et al., 2000; Ortmanns et al., 2007). The electrode–tissue interface can be equivalent to a model with capacitance and resistance. The cathodic stimulation is used for AP triggering and the anodic stimulation is used for charge compensation. In Figure 2B, the intermediate delay ensures the transfer of AP. The bidirectional current with high matching is required to ensure that the nerve tissue has no charge accumulation to avoid nerve tissue damage.

Though the CCS has become the most common method, the stimulation voltage is greatly affected by the electrode impedance, especially in multi-channel stimulation cases. The supply voltage needs to ensure the minimum necessary voltage level applicable for various loads, to achieve the required power efficiency. Other stimulation methods such as switched-capacitor stimulation (SCS) can control the amount of stimulus charge better and achieve higher power efficiency. However, it is difficult to integrate the large capacitors into the chip. Recently, the feasibility of high-frequency stimulation has been proved (van Dongen et al., 2015), and a high-frequency switched capacitor stimulation (HFSC) method has been proposed in Hsu and Schmid (2017). As shown in Figure 2D, a method using high-frequency switching for stimulation is introduced. Due to the small amount of charge transferred in each switching process, the required capacitance C_stim_ can also be reduced exponentially, which is convenient for on-chip integration and reduces the chip area and cost. The switching timing and the voltage waveform of the electrode (V_E_) are shown in Figure 2E. The phase difference is introduced between S1 and S2 to remove the dead zone. V_E_ increases with the number of switching, and AP will be triggered when the charge accumulation reaches the threshold.

During the stimulation process, the bidirectional current cannot match completely, which causes the residual charge in the nerve tissue. The accumulation of residual charge will cause irreparable damage to the nerve tissue. Considering the safety of neural stimulation, the designed stimulator requires minimum residual charge in a single cycle, and the accumulated charge after multiple cycles also needs to be removed in time. The real-time monitoring of V_E_ is necessary to eliminate the residual charge in time when the voltage does not return to the reference voltage at the end of the stimulation cycle (Ortmanns, 2007). A variety of the accumulated charge balance methods are introduced, such as the electrode short-circuit technology (Rothermel et al., 2009) and the short-time pulse insertion technology (Ortmanns et al., 2007; Yao et al., 2015; Chen et al., 2020). In Figure 2C, the electrode short-circuit technology uses switch S to connect the electrode and ground. This method will produce unpredictable discharge time, which depends on the electrode impedance. As for the short-time pulse insertion technique, it can achieve controllable compensation. As shown in Figure 2F, when V_E_ is detected to be out of the reference voltage range at the end of each stimulation cycle, a series of short-time pulses will control the switch for charge compensation to recover the V_E_ voltage level (Sooksood et al., 2010). However, the frequent short-time pulse stimulation will introduce more switching noise and reduce the signal-to-noise ratio (SNR) of the recorded signal.

A cooperative compensation method is proposed (Butz et al., 2018) to ensure that the residual charge is unable to damage nerve tissue under long-term stimulation by using the “cause-based and consequence-based systems.” As shown in Figure 2H, the stimulation mode is CCS in this method. The consequence-based system is named Inter-Pulse Charge Control (IPCC) due to its instantaneous compensation properties between the stimulations. When the voltage V_E_ changes greatly at the end of the stimulation cycle, the high-voltage output stage of the IPCC will generate a constant compensation current and compensate the residual charge until V_E_ returns to the reference voltage. The cause-based compensation method is called offset compensation (OC). A stable feedback system is introduced through the PI control, and the compensation will be performed in the next stimulation cycle. When the OC is working, the PI control will add extra bias current to the cathode current. In the next stimulation cycle, the accumulated charge would be compensated by the improved bidirectional current. After each bidirectional current stimulation, the two compensation methods work independently using S1 and S2 control, which avoids disturbance caused by simultaneous sampling. As the OC time is shorter, the voltage sampling should be finished before the IPCC starts working.

In addition, the power efficiency of the stimulator is also an important design consideration, as higher power efficiency means less thermal power consumption. Excessive thermal power generation will not only cause nerve tissue damage, but also affect the working environment of the stimulator. A new type of adiabatic current-controlled stimulator architecture is adopted in Ha et al. (2019). Under the condition of ensuring better power efficiency, a complete wireless power supply is realized. The adiabatic stimulator can track the change of V_E_ and minimize the voltage drop across the current source. The adiabatic waveforms are provided by the on-chip resonant coil, which are directly synthesized by cascading and folding auxiliary rectification stages under the demand of stimulating voltage. In addition, the whole circuit is improved with better energy-saving performance by realizing the function of recovering electric charge from nerve tissue. The process is shown in Figure 2G. The stimulation is supplied by V_DD–stm_ and V_DD–stm_ at first. After the AP is triggered, reverse current compensation is carried out. In the second stage, the charge inflows to V_DD_ and V_SS_, which delivers the energy back to the supply rails. Compared with the traditional methods which draw the charge down to a negative power supply or ground, this method prevents energy loss in the stimulation process and improves power efficiency.

Table 1 shows the comparison of the important parameters in the design of neural stimulation circuits. It can be seen that the CCS is still the main stimulation type for the neural stimulators, because the charge injected into the tissue during stimulation can be controlled using CCS. For the power supply, the implantable stimulator requires a wireless power supply with inductive link, while the external wearable stimulator uses a battery. The stimulation safety and energy efficiency are important for neural stimulator design. It is necessary to monitor and remove the residual charge remaining in tissue on time through the voltage detection circuits and the pulse injection circuits or other circuits with better current matching. The performance of peak efficiency refers to the ratio of the maximum output power to the power supply. The highest peak efficiency is 80% among the listed prior works. The maximum stimulation current represents the strength for neural or muscular stimulation. Considering the stimulation effectiveness, most stimulators have the maximum output current not less than 1 mA.

**TABLE 1:** Comparison of the parameters in neural stimulation circuits.

	Hsu and Schmid, 2017	Ha et al., 2019	Butz et al., 2018	Noorsal et al., 2012	Lee et al., 2015	Sooksood et al., 2010	Lee et al., 2013	Song et al., 2012
Technology (nm)	HV180	180	350	350	350	PCB	500	130
Stimulation type	HFSC	CCS	CCS	CCS	SCS	CCS	CCS	CCS
Supply voltage (V)	5	0.8	22	20	4	30	5	3.3
Power source	Battery	Inductive link	Battery	Battery	Inductive link	Battery	Inductive link	Battery
Safe voltage detection	N/A	Current matching	IPCC/OC	OC/Short pulse injection	Charge monitoring	OC/Short pulse injections	Charge monitoring	Current matching
Safety window (mV)	N/A	–	±100	±100	N/A	±100	±50	N/A
Peak efficiency (%)	49	63.1	–	62	80.4	–	68	80
Maximum stimulation current (mA)	N/A	0.145	5.12	1	4	1	2.48	1
Power/CH (mW)	0.063	–	11	1.16	–	–	3.75	6.8
Area/CH (mm^2^)	0.035	0.0484	1.5	0.2	3	N/A	0.3	1.25

### Neural Recording

**FIGURE 3:**
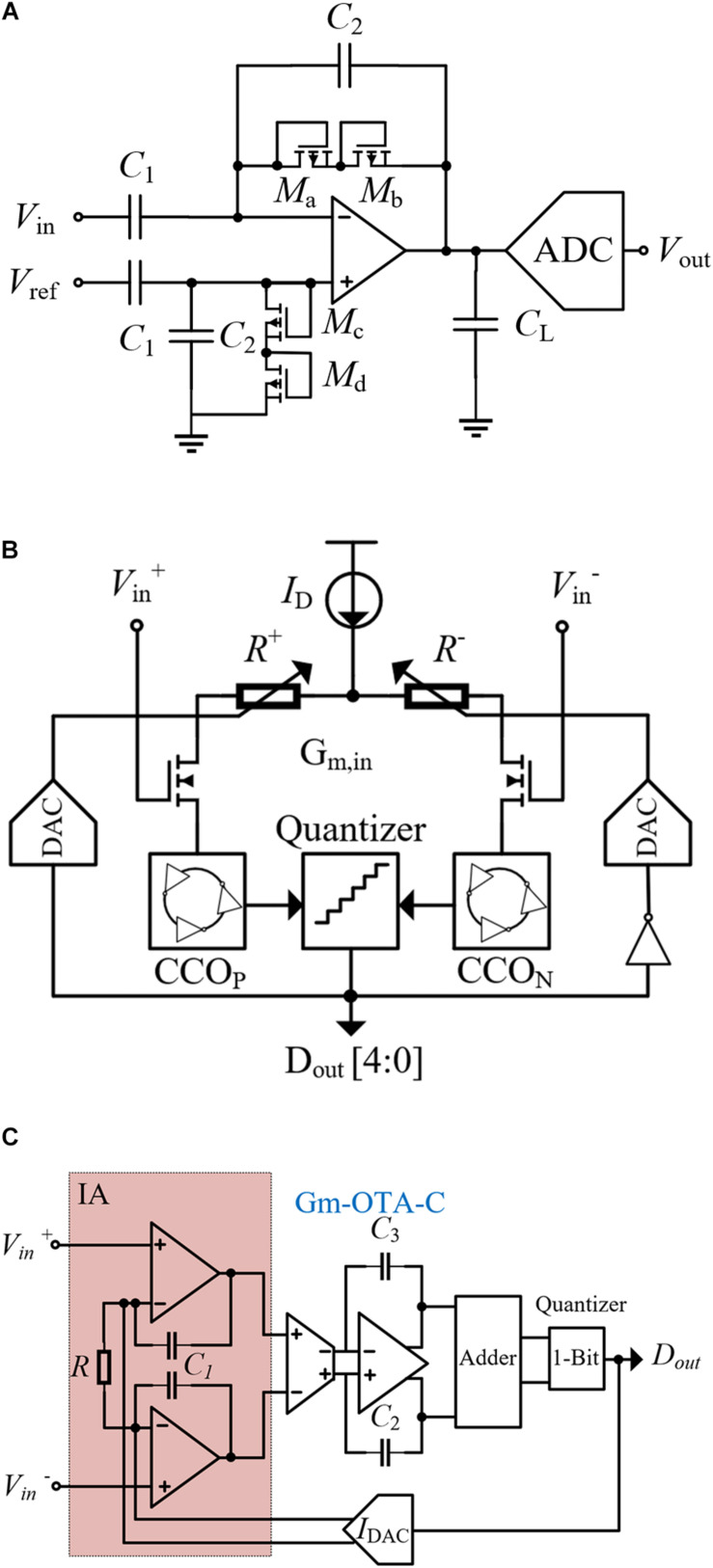
**(A)** Traditional structure of the neural recorder. **(B)** Structure of the VCO-based neural recorder. **(C)** Structure of the neural recording circuit based on CTDSM.

In a closed-loop neural system, in addition to the stimulator that triggers the AP, neural recording is required to sample local field potentials (LFPs). If the stimulator is regarded as the executor, the neural recording part is the digital back end of the whole system. Different from stimulation signals, LFPs are the electric potentials in the extracellular medium around neurons, which have very small amplitude (μV) and low frequency (1–200 Hz). Due to the microvolt level of the nerve signal, it is not reliable to implement direct digital quantization before amplifying. The most common way is to add a low noise amplifier (LNA) to the front end of the recording (Harrison and Charles, 2003; Harrison et al., 2007) and then to add the digital quantization circuits (Zou et al., 2009; Liu et al., 2017; Yu et al., 2018). As shown in Figure 3A, the recording is completed by the cooperation of the LNA and analog-to-digital converter (ADC). The gain of the amplifier is determined by the feedback capacitor C_2_ and the input capacitor C_1_, and C_L_ is the load capacitance. The PMOS transistors with diode connection (M_a_–M_d_) act as pseudo resistors. Besides, a high-pass filter (HPF) with low cutoff frequency is formed with input capacitors. The HPF is used to eliminate the DC offset in neural signals to prevent recording saturation.

In the traditional methods, the HPF is used to block the DC offset by using the input in an ac-coupled form. However, due to the information at low frequency carried by nerve signal, the HPF needs a large input capacitance, which reduces the input impedance of the neural recording circuit. In Jeon et al. (2019), a neural-recording IC using a voltage-controlled oscillator (VCO) is proposed, which can quantize the input signal directly and achieve a high dynamic input range. The circuit structure of the recording is shown in Figure 3B. This method is adopted and converts voltage into differential currents by an ac-coupled input transconductance circuit. Then, the current is fed into the current control oscillators (CCOs) CCOP and CCON. According to the phase difference between CCOP and CCON, the quantizer generates the digital output D_out_. The digital-to-analog converter (DAC) controls the input resistance through a negative feedback, and reduces the difference between I_in__+_ and I_in__–_. Due to this negative feedback action, the values of I_in__+_ and I_in__–_ assume almost the same value even if the input voltage is large, which results in a wide input linear range. In addition, the input of the circuit is directly connected to the gate of the MOSFET, which has a large input impedance and improves the recording stability.

Another new architecture uses a continuous-time delta-sigma modulator (CTDSM) as the recording front end (RFE), and the researcher establishes the structure based on a second-order CTDSM (Nikas et al., 2019). As shown in Figure 3C, it has a cascaded integrator and a feedforward compensation architecture with distributed feed-in paths and a single-bit quantizer. The first integration stage is realized by using an improved instrumentation amplifier (IA), and the second integrator is implemented with the G_m_-C-OTA circuit. The feedforward branches are summed up by the switched capacitor adder, and the quantizer generates digital signals. The feedback IDAC adjusts the bias current of the IA and improves the stability of the first stage. Similar to the way of using voltage-controlled oscillators, the input of the recording is directly connected to the gate of the MOSFET, showing a large input impedance. In addition, both the VCO and CTDSM use quantizer output and feedback DAC modulation, which improves the linear input range of the circuit. The difference recording method has large-signal common-mode rejection. In fact, with the growing demand for neural recording, such as monitoring nerve signals from hundreds of electrodes at the same time, it is necessary to realize intelligent data acquisition systems in the case of low power consumption and small area. Due to the various interface impedance caused by the differences in electrode sizes and materials, a high input impedance front end for neural recording is required. Currently, the recording method based on the gain stage and ADC is gradually replaced. On the contrary, direct conversion to analog front end (AFE) has the advantages of high input impedance, low power consumption, and small area, which would become the future development direction.

Table 2 shows the comparison of neural recording circuits. According to the recorded signals, the designed bandwidth is different. LFP occupies a low-frequency band from 1 to 200 Hz, and AP occupies a higher frequency band from 200 Hz to 10 kHz. The peak input refers to the linear input range of the neural recording, which limits the maximum input range of the circuit. The input-referred noise (IRN) affects the quality of the neural recording. The SNR can be improved by reducing the IRN. To prevent the attenuation of the neural signals, the input impedance (Zin) of the neural recording circuit must be significantly greater than the electrode impedance (Zin > 1G), and the DC current of the electrode should be limited within 100 pA. The high-gain LNA (>40 dB) may cause poor artifact tolerance, as the large-scale artifacts would cause saturation of the amplifier.

**TABLE 2:** Comparison of the parameters in neural recording circuits.

	Jeon et al., 2019	Samiei and Hashemi, 2019	Nikas et al., 2019	Zou et al., 2009	Shen et al., 2018	Park et al., 2018	Muller et al., 2015	Jiang et al., 2017	Chandrakumar and Markovic, 2018
Technology (nm)	180	180	180	350	180	180	65	40	40
Supply voltage (V)	1.2	0.6/1.2	1.8	1	1.0	0.5/1.0	0.5	0.45/1.2	1.2
Target application	LFP	AP and LFP	LFP	LFP	AP and LFP	AP and LFP	LFP	LFP	AP and LFP
Peak input (mV)	200	–	208	5	–	3	±50	±50	200
Input referred noise (μVrms)	1.3	3.2/2.0	2.3	2.5	5.5	3.32	1.3	5.2	6.35
Zin (Ω)	0.16G	3.0G	∞	–	∞	∞	28M	∞	1.5G
Gain (dB)	N/A	41–59	N/A	60	25.6	37.5–52.9	N/A	N/A	18
Bandwidth (Hz)	200	0.5–5k	250	0.005–292	4–10k	0.4–10.9k	1–500	1–200	1–5k
Power/CH (μW)	3.9	2.6	23	0.895	0.25	1.22	2.3	7	7.3
Area/CH (mm^2^)	0.225	0.08	0.694	1	0.29	0.05	0.025	0.135	0.113

In neural recording, the artifact-induced problem of stimulation sometimes emerges and affects the function of biomedical devices for brain stimulation and recording (Asfour et al., 2007; Chen et al., 2011; Lin et al., 2013; Caldwell et al., 2019). As shown in Figure 1B, in the closed-loop neural recording and stimulation circuit for epileptic seizure detection and suppression, the stimulator is triggered, and it generates stimulation pulses in certain regions of the brain to suppress the epileptic seizure when an epileptic seizure episode is detected from the intracranial electroencephalogram (iEEG). However, the large stimulation pulse causes the artifact that is subsequently picked up by the recording amplifier as a false AP, and a false stimulation will be triggered. This situation is even worse in the multi-channel neural recording and stimulation circuit (Ng et al., 2012; Joseph et al., 2018).

The detailed artifact origin and the corresponding artifact-removal techniques are presented in the next sections.

## Key Issues in Neural Recording and Stimulation Circuits

### Stimulation Induced Artifact in the Closed-Loop System

Most neural/muscular recording and stimulation circuits in biomedical devices consist of multiple recording and stimulation channels, AP detection and data processing circuits, stimulation circuitry, and electrodes. During the operation, the large stimulation current causes the tissue potential to change and the tissue potential fluctuation will propagate to the recording site and cause artifacts (McGill et al., 1982). For bipolar stimulation, there are two stimulation electrodes, namely, a working electrode and a reference electrode. During the stimulation, most of the biphasic current flows between the working and the reference electrodes through the stimulated tissue. In the cathodic phase, the electric potential near the working electrode decreases since the stimulator sinks current from the reference electrode. While in the anodic phase, the electric potential near the working electrode increases since the stimulator generates current to the reference electrode through the tissue–electrode interface. The amplitude of this voltage variation is usually from several hundred millivolts to several volts (Xu et al., 2017), which depends on several factors, including the electrode impedance and the power-supply voltage at the output stage of the stimulator. The voltage variation would also be recorded by the neural recording circuit and cause saturation of the recording amplifier, which produces the artifact (Johnson et al., 2017; Jeon et al., 2019). Such a stimulation artifact can be observed in most of the closed-loop recording and stimulation circuits (Yoshida and Horch, 1996; Blum et al., 2007; Venkatraman et al., 2009; Mc Laughlin et al., 2012; Ng et al., 2012; Xu et al., 2012). The amplitude of the recorded artifact spike is determined by several factors such as the distance between the recording and the stimulation sites, the gain of the amplifier, and the electrode impedance (Johnson et al., 2017; Pazhouhandeh et al., 2018; Jeon et al., 2019; Uehlin et al., 2020). The artifact is typically hundreds of millivolts in amplitude, and 10 to 100 times higher than the amplitude of the recorded neural signals (Dabbaghian et al., 2019; Lee and Je, 2020).

**FIGURE 4:**
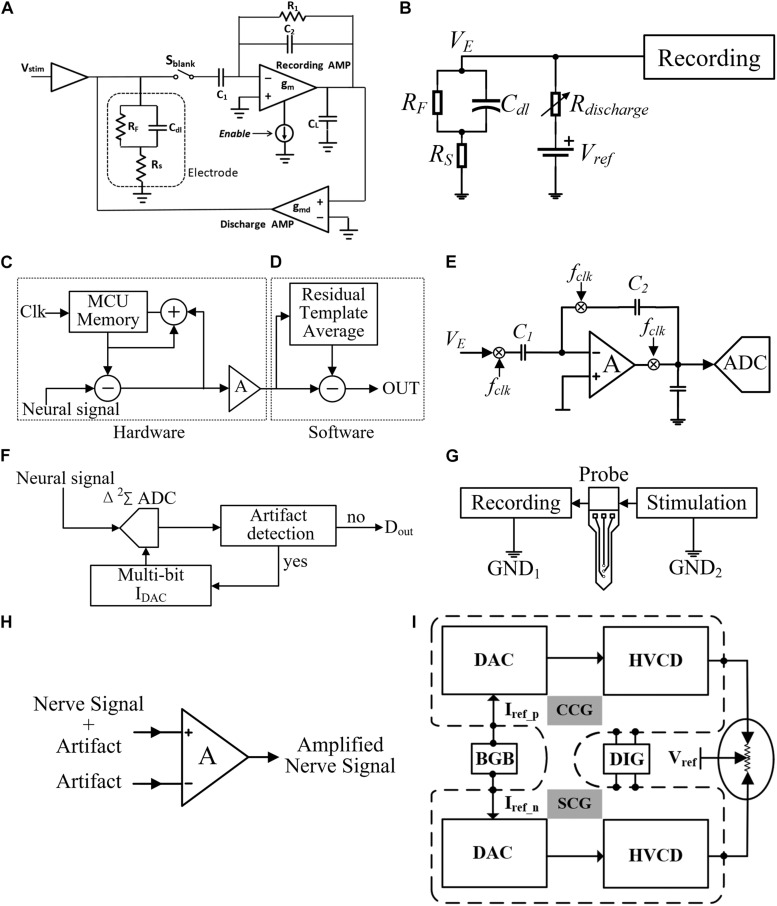
**(A)** Artifact elimination circuit with blanking technique, **(B)** active electrode discharge technique, **(C)** iterative hardware loops, **(D)** adaptive filtering technique in post-processing, **(E)** chopper technique, **(F)** track-and-zoom (TAZ) neural ADC, **(G)** localized stimulation technique, **(H)** dual electrode in-phase stimulation, and **(I)** RTPPS technique.

Several stimulation artifact cancelation techniques have been reported previously. The blanking technique and digital signal processing (Erez et al., 2010) have been used to cancel the artifact (Olsson et al., 2005; Venkatraman et al., 2009; Kent and Grill, 2011; Myers et al., 2011; Zoladz et al., 2012; Wei-Ming et al., 2013; Yi et al., 2013; Bozorgzadeh et al., 2014; Elyahoodayan et al., 2019). In the blanking technique, the RFE is switched off or disabled (input is shorted to ground) during the stimulation period and turned on after the stimulation is completed to continue the recording. As shown in Figure 4A, the recording amplifier and two capacitors (C_1_ and C_2_) are used to amplify nerve signals. A very large resistor R_1_ is used in the feedback path to provide a DC current path to bias the input. The discharge amplifier helps the electrode return to its pre-stimulation voltage after stimulation. The recording amplifier is disabled through S_blank_ during stimulation and enabled after 2 ms when the stimulation ends (Blum et al., 2007). This method is effective in some applications, such as EMG signal acquisition. Because the evoked neural spike usually emerges with a latency, the AP and the artifact spike will not overlap. However, in some other applications, such as neural prosthesis or deep brain stimulation (DBS), the neural responses in the cathodic and anodic stimulation phases also need to be recorded. In such applications, if the blanking technique is employed, the neural signals during the “blanking” period cannot be recorded and thus some important neural information may be missed.

The artifact cancelation using digital signal processing can be divided into two categories: real-time signal processing and signal post-processing, such as active electrode discharge in real-time signal processing (Brown et al., 2008). As shown in Figure 4B, R_discharge_ is a variable resistance and the impedance is very large under normal conditions. When a large artifact is detected, R_discharge_ can be changed to a value with low impedance, then the RC time constant of the path is reduced and thus it makes the electrode voltage quickly return to the reference voltage. This method reduces the recovery time from 10 ms to approximately 200 μs, but the problem of artifact recording has not been solved. In signal post-processing, the recorded neural signal together with the artifact are acquired. One processing method is adaptive filtering (Mahajan and Morshed, 2015; Rozgic et al., 2019; Samiei and Hashemi, 2021). The template of the artifact waveform can be obtained by the least-mean-square algorithms, genetic algorithms (Qiu et al., 2015), principal component analysis (Deprez et al., 2017), and wavelet algorithms (Yochum and Binczak, 2015). As shown in Figure 4D, the neural signal can be recovered by subtracting the artifact template from the collected signal. One disadvantage of signal post-processing is that the RFE must have a large dynamic range so that the artifact does not saturate the amplifier. The merit of removing artifacts using digital processing compared with blanking is that no neural spikes are missing in the recording. However, the digital processing is computationally intensive. The artifact templates produced by different tissue parts are also inconsistent. Some improved schemes have been proposed in Culaclii et al. (2018), in which both hardware and software are implemented to optimize the system. As shown in Figure 4C, the amplitude of artifacts is reduced by iterative hardware loops instead of filtering them completely. The hardware loop stores the artifact as the template and then iteratively updates the template according to the recording difference, until the template converges within the resolution range of the hardware component. Finally, the artifacts are removed by signal post-processing. However, the hardware implementation may introduce the noise caused by other electronic components from the PCB boards. Besides, the software implementation also consumes extra computation resources.

At present, all kinds of signal post-processing methods have become mature. The research focus on artifact suppression has been changed to establishing a high input dynamic range RFE, removing the amplifier saturation caused by artifacts. A common combination composed of a chopper amplifier and the ADC is proposed in Chandrakumar and Markovic (2017; 2018) and Samiei and Hashemi (2019; 2021). As shown in Figure 4E, chopping is an effective way to reduce the low-frequency flicker noise of the amplifier. The gain of the capacitive feedback amplifier is determined by the ratio of C_1_ to C_2_. To eliminate the influence of the operational amplifiers’ low-frequency noise and the DC offset, the chopper is used to up-convert the low-frequency biological signal to the carrier frequency (F_clk_), away from the DC offset and flicker noise. After band-pass amplification, the up-converted signal is down-converted to its original frequency, and the DC offset and flicker noise are up-converted away from the signal. However, due to the large input capacitor C1, the input impedance is restricted.

A track-and-zoom (TAZ) neural ADC is proposed in Reza Pazhouhandeh et al. (2020). As shown in Figure 4F, a recording amplifier and an ADC are combined. When fast artifact transients are detected, the multi-bit DAC will feedback to the TAZ ADC. Then, the dynamic input range of the RFE is exponentially expanded, which prevents the saturation of neural recording and saves chip area and power consumption. In another innovative method, the recording amplifier is replaced by VCO (Jiang et al., 2017; Jeon et al., 2019). As shown in Figure 3B, the proposed circuit in this method quantizes the frequency of the sample by counting the phase increment. Applying this method, the neural signals recording can be done in the frequency domain. The feedback DACs compensate the nonlinearity of G_m,in_. Thus, VCOs can keep good linearity in a large input range of neural recording. However, in order to ensure high sensitivity of recording with large input range, the noise of VCOs dominated by flicker noise needs to be further reduced.

Another artifact suppressing technique reported is the localized stimulation (Wong et al., 2007; Yung-Chan et al., 2009), as shown in Figure 4G, where the stimulation current returns to a local ground. Although this reduces the artifact amplitude at the input of the recording amplifier and allows the amplifier to quickly recover to the normal state, the artifact is still not effectively suppressed. An improved method is shown in Figure 4H. The dual-electrode in-phase stimulation and differential acquisition at the recording electrodes are carried out (Nag et al., 2015). This method uses the common mode suppression characteristics of a differential input to reduce the artifacts. The experimental result showed good artifact suppression effect, but this method requires the electrode impedance to be highly matched. In order to ensure the consistency of the common-mode level, it is necessary to establish an additional accurate impedance matching network.

To avoid impedance mismatching in differential acquisition at the recording electrodes, the referenced and tuned push–pull stimulation (RTPPS) scheme with a tri-polar electrode is proposed (Xu et al., 2017). The problem of the artifact can be solved and no blanking of the recording channels is needed. As shown in Figure 4I, the RTPPS uses a tri-polar stimulation configuration with two working electrodes and one reference electrode. The stimulation currents delivered by the two working electrodes are complementary to each other. By doing so, the amplitude of voltage fluctuation at the recording site can be significantly reduced.

Several other artifact cancelation methods have also been proposed. In Liu et al. (2011), neural recording is carried out only in the mid-phase between cathodic and anodic stimulation phases to avoid the artifact. In Dura et al. (2012) and Chu et al. (2013), high-frequency short-duration pulses or other specific patterns are adopted for stimulation. However, the stimulation parameters (i.e., pulse width, amplitude, and frequency) are usually determined by the application and not by the artifact cancelation.

Artifact is a key issue in neural recording. Table 3 compares various methods of artifact suppression. These methods can be divided into two categories. In the first category, the artifact suppression is done at the RFE by using optimized neural recording circuits, while in the second category, the artifact suppression is implemented by using the digital signal processing after recording. The methods of suppression at the RFE can reduce the maximum artifact amplitude of neural recording and reduce the design complexity. The methods using digital signal processing after recording rely on different algorithms (adaptive filtering, etc.). By comparing the prior works in Table 3, it is found that the method (Hardware and Software) using both RFE optimization and digital processing can achieve the highest artifact suppression ratio (100 dB).

**TABLE 3:** Comparison of the artifact suppression methods.

	Samiei and Hashemi, 2021	Xu et al., 2017	Culaclii et al., 2018	Pazhouhandeh et al., 2018	Uehlin et al., 2020	Rozgic et al., 2019	Reza Pazhouhandeh et al., 2020	Jiang et al., 2017
Technology (nm)	180	180	N/A (SOC)	130	65	40/HV180	130	40
Supply voltage (V)	1/3	1	5.25	1.2/3.3	1.2/2.5	0.6/1.2/1.8	0.6/1.2/3.3	0.45/1.2
Artifact suppression	FE filter	RTPPS	Hardware and software	Differential Acquisition	Digital adaptive filter	Online adaptive filter	Track-and-zoom	Direct digitization
Bandwidth (Hz)	200–9k	200–5.8k	1–10k	–	<32k	1–250	1–500	1–200
ADC type	SAR	N/A	–	Δ	Nyquist Δ-Encode	–	Δ^2^Σ	SAR
ADC ENOB (bit)	8.6		–	9.7	14	12.8	11.3	12.0
Maximum tolerated artifact (V)	0.7	1	5	0.01	0.11	0.1	0.2	±0.05
Artifact suppression ratio (dB)	–	30	100	78	60	42	N/A	N/A
Power/CH (μW)	4.3	2.4	–	0.73	0.62	8.2	4.913	7
Area/CH (mm^2^)	0.66	–	N/A	0.0054	0.0025	0.12	0.023	0.135

### Probes in Neural Recording

**FIGURE 5:**
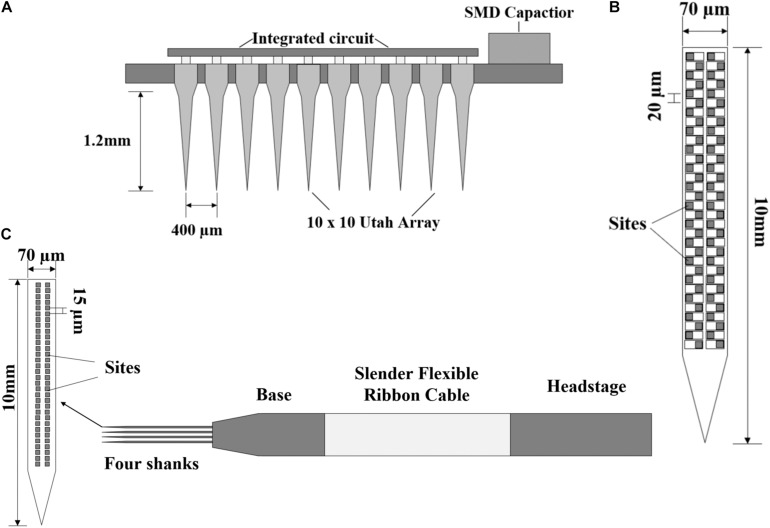
**(A)** The structure of a 100-electrode neural recording circuit with Utah array. **(B)** Neuropixels 1.0 probe with the sites arranged in a four-column checkerboard. **(C)** Structure of Neuropixels 2.0 probe with the sites arranged vertically in two columns.

The purpose of neural recording is to record the activities of neurons; however, how to record a large number of neurons in multiple regions for a long time is a key issue. The implanted probes must contain multiple electrode arrays and ensure the reliability of long-time recording. A 100-electrode neural recording circuit with a Utah probe is proposed in Harrison et al. (2007), and the probe design is shown in Figure 5A. This Utah probe uses a 10 × 10 array of platinum-tipped silicon extracellular electrodes. The silicon-based electrodes were inserted into the cerebral cortex and the researchers can record the electrical activities of nearby neurons. The flipped chip is connected to all 100 electrodes through the back of the Utah array, and it can sample in a plane approximately parallel to the brain surface.

However, the best way to record in layered or deep structures (striatum, hippocampus, or superior colliculus) is to take a dense sample in a plane perpendicular to the brain surface. A breakthrough development named Neuropixels probe is proposed (Jun et al., 2017). This is the first report of a large (10 mm) dense (100 sites per millimeter) implantable nerve recording device. The structure of the probe is shown in Figure 5B. The 12 × 12 μm sites are arranged in a four-column checkerboard and 20 μm center-to-center nearest neighbor spacing. The probe is 10 mm long and contains 960 sites. In addition, it has a user-programmable switch that allows us to address 384 of the 960 sites simultaneously. Titanium carbide (TIN) is selected as the recording site material, which is compatible with CMOS processing and has the advantages of low and uniform impedance. The researchers used two Neuropixels probes to record the activities of more than 700 neurons. This combination of high-performance electrode technology and scalable chip manufacturing opens a way to record the brain-wide neural activities and neuron behaviors.

Chronic recording is essential for understanding the processes that evolve over time, such as learning, memory, and plasticity. In the latest research, the problem of stable recording of a single neuron on a long-time scale from several weeks to several months has been overcome (Steinmetz et al., 2021). Based on Neuropixels 1.0, the team has successfully developed a more miniaturized Neuropixels 2.0 with more recording sites. The electrode structure is shown in Figure 5C. The probe consists of four slices inserted into the brain and a probe base (the voltage signal is filtered, amplified, multiplexed, and digitized on the base). The weight of the probe plus a headstage is about 1.1 g. The base is fixed on a rigid printed circuit board (PCB) and a slender flexible ribbon cable that plugs into a headstage. Compared with the 20 μm of Neuropixels 1.0, the recording sites are arranged vertically in two columns rather than staggered, and the vertical distance from center to center is 15 μm. The length of the probe is still 10 mm and the number of recording sites per handle is 1,280. The four-shank version supports 5,120 recording sites and the headstage is miniaturized to about one-third of the size of those for Neuropixels 1.0, which is more suitable for chronic recording in a freely moving mouse. Using two four-shank probes, combined with a motion correction algorithm, the success rate of neuron tracking is more than 90% in 2 weeks and more than 80% successful for up to 2 months. These experimental data are acquired based on chronic recording, and the results prove that the proposed recording electrode (Neuropixels 2.0) is very suitable for brain recording with stable insertion and contact.

### Wireless Power Supply and Wireless Communication

**FIGURE 6:**
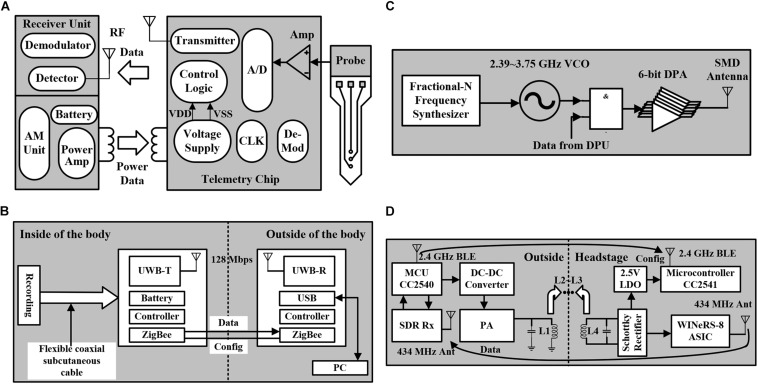
**(A)** Traditional structure of the wireless powered neural recorder. **(B)** Block diagram of the neural recorder using the UWB transmitter for BMI. **(C)** The block diagram of an energy-efficient wireless transmitter using dual-band on–off keying modulation. **(D)** The block diagram of the four-coil inductively powered wireless neural recording and stimulation system.

In the system-level design of neural stimulation and recording, another necessary module is the power supply. To achieve a complete closed-loop system and satisfy the needs of large-scale electrode array such as the applications in the brain and wireless power supply is necessary for implantable neural chips. The traditional wireless power supply design is shown in Figure 6A, which introduces an inductive RF telemetry link (Wise et al., 2004). The outside part includes the receiving unit and the driving unit. The power supply of the implantable chip is provided by the inductors. The neural recording requires multiple channels, and each channel supports several recording sites. The recorded neural signal is converted into a digital signal by the ADC, and transmitted to the outside receiver unit through an RF link. The clock needed for the implantable chip is generated from the RF carrier. The demodulator is used to decode the received data. Then, the data can be transmitted to the computer for storage.

Recent research in nerve prosthesis chips demands high-quality data transmission from multiple neural electrodes. When the data throughput is large in multiple-channel neural recording, wireless transmission is needed as it can reduce the number of connected wires and simplify the interface. A multi-channel neural recording for brain–machine interface (BMI) is proposed (Ando et al., 2016), which adopts an ultra-wideband (UWB) transmitter. The high transmission rate ensures the stable recording of multi-electrodes, and the low output power has stronger anti-interference ability. In addition, the neural recording module and the wireless data transmission module are implanted in the brain and abdomen, respectively. The separated modules are connected by a flexible coaxial subcutaneous cable, which can transmit high data rate signals. The structure of the system is shown in Figure 6B, achieving a transmission rate of 128 Mbps, which is enough to support the application of thousands of electrodes. However, the system also has some limitations. The ZigBee module is added to control the implant because the communication direction of UWB can only be unidirectional (from inside to outside). The communication range is only 20 mm, which means that the external communication device must be carried at any time. Besides, the space for implantation is constrained due to the short range.

In the application of multi-electrodes, to break the limitation of power efficiency, the transmitter needs to have a high energy efficiency. Recently, a high energy-efficient wireless transmitter using dual-band on-off keying modulation has been proposed (Lyu et al., 2020), which supports 2.4-GHz and 3.2-GHz bands. The structure of the transmitter is shown in Figure 6C, including a fractional-N frequency synthesizer and a 6-bit Class-D digital power amplifier (DPA). The frequency range of the VCO is from 2.39 to 3.75 GHz, covering the working frequency band of the transmitter. The output carrier of the synthesizer is modulated by the coded data from the data processing unit (DPU). Then, the data are sent to the DPA for amplification, and finally transmitted to the SMD antenna for transmission. This structure is based on the interfacing system-on-a-chip (SoC) mode, which achieves a transmission rate of 54 Mbps and a transmission distance of 4 m. Due to the reservation of the wired communication for SPI interface, the circuit can only transmit unilateral wireless data (from inside to outside), which is not applicable for implantation.

Due to the lack of sufficient power budget and two-way wireless communication, most of these devices only support independent neural recording or stimulation function. It is necessary to combine the two functions. A SOC with four-coil inductive power supply is proposed (Lee et al., 2019), which integrates 32-channel neural recording and 4-channel stimulation circuits, and achieves a data transmission rate of 434 MHz. As shown in Figure 6D, the four-coil induction power supply mode improves the power efficiency, and the CC2540 micro-controller unit (MCU) connects the DC–DC converter to realize power control. SDR Rx is the external software-defined radio wideband receiver, and PA is a power amplifier. In addition, the MCU sends stimulation parameters and setting parameters to the headstage through the BLE link. The headstage includes a WINeRS-8 ASIC, a Schottky rectifier, an RX MCU (CC2541), and a 2.5-V LDO. The WINeRS-8 ASIC consists of 32-channel neural recording and 4-channel CCS circuits. Because the BLE link does not have enough transmission rate to meet 32-channel applications, a 434-MHz OOK transmitter is added to transmit AFE recorded data. The system integrates the functions of nerve recording and stimulation and achieves a high data transmission rate. However, the four-coil power supply mode limits its application, which can only be used in an energy cage formed by four coils. The BLE link is not based on peer-to-peer communication, but on multiple inputs to multiple outputs mode, which might be vulnerable to radio interference.

The implanted neural chip requires a wireless power supply and a wireless communication system. Table 4 shows the comparison of the parameters of the circuits with wireless power supplies. The power frequency refers to the frequency of the alternating current (AC) of the induction link, and the external power supply is generally realized by using the inductive coils. The transmission distance is related to the size of the inductive coil, and the transmission distance ranging from 15 to 20 cm can be achieved in the prior works. According to the comparison in Table 4, only a few designs incorporate both the function of neural recording and stimulation in one implanted neural chip. The uplink data and downlink data are related to the data transmission mode between internal and external. With the increase of the number of channels, the data transmission rate also needs to be improved consistently.

**TABLE 4:** Comparison of the parameters of wireless power supplies.

	Piech et al., 2018	Jia et al., 2017	Mei et al., 2017	Lee et al., 2019	Kassiri et al., 2016	Lee et al., 2016	Lo et al., 2016
Power frequency (Hz)	1.85M	13.56M	346.6M	13.56M	1.5M	13.56M	2M
Distance (cm)	0.5	20	18	18	15	18	–
Coupling	Ultrasound	4 coils	3/4 coils	4 coils	2 coils	3/4 coils	2 coils
Recording	N/A	N/A	EEG	Spike	EEG	Spike	EMG
Stimulation	CCS	CCS	N/A	CCS	CCS	N/A	CCS
Uplink data	LSK	BLE	2.4 GHz RF	OOK/BLE	UWB/FSK	FSK	LSK/WiFi
Downlink data	OOK	BLE	N/A	BLE	ASK	N/A	DPSK/WiFi
Area (mm^2^)	3.1 × 1.9 × 0.89	20 × 22 × 11	14 × 25 × 14	19 × 19 × 30	20 × 20	25 × 35 × 8	4.4 × 5.7
Power (mW)	0.15	43	6.4–13	35	6.9	51.4	–
Experiment area (cm^3^)	N/A	20 × 46 × 20	61 × 61 × 30	24 × 46 × 20	26 × 45	30 × 28 × 18	0.5 × 0.5 × 0.5

## Discussion

Since several advances in neural recording and stimulation integrated circuits are introduced in this article, it is worthwhile to present a discussion about key indicators for the design, which will help the circuit designer improve the chip performances.

In the design of neural stimulators, the important parameters are safety and efficiency. The essence of stimulation is the injection and recovery of electric charge. For fragile nerves, excessive injection of electric charge will cause irreparable damage. Therefore, we need to restrict the stimulation current and reduce the influence of residual charge in the tissue. CCS is still the mainstream design for neural stimulation. In recent years, the combination of multiple control methods to reduce the residual charge has gradually become the mainstream. As for efficiency, the electrode voltage is highly dependent on the electrode impedance, so more energy loss will be generated. The generation of large energy in the form of heat could be harmful for the tissue around the implantable neural chips. How to effectively reduce heat generation is still an issue for implantable chip design.

For the design of neural recording, accuracy is a key parameter. Due to the small amplitude and low-frequency characteristics of neural signals, the difficulty of sampling is greatly increased. In addition, the impact of the stimulation artifact, the attenuation of neural signals, and the crosstalk of electrical signals are needed to be further researched. There are several irrelevant signals in the collected signals that are difficult to filter out. In the design process, both front-end processing and back-end adaptive filtering are the common ways to solve the problem. In the latest technology, the direct optimization of the AFE has the advantages of achieving high input impedance, high dynamic range, low power consumption, and small area, which would become the future development direction.

At present, the diversity and miniaturization of neural recording and stimulation circuits is a trend. For the requirement of chip implantability, due to the large volume of wired power supply, the application of wireless power supply is necessary. Through wireless transmission, the recorded data are transmitted to the computer terminal, and then the terminal transmits stimulation instructions back after computation. For multi-electrode recording demands, the transmission rate and transmission distance of wireless communication also need to be improved. The trade-off between area and power needs to be carefully considered for different applications. In addition, as the common-mode voltage affects the nerve signals recording, the technique of stimulation artifact suppression is still important to be further researched. The current solution could be divided into two aspects. One is the artifact suppression of the RFE, such as iterative hardware loops or RTPPS technology. The other is to sample the artifact signal followed by filtering or digital post-processing to get the complete neural signal at the neural recording site. The combination of the two schemes for artifact reduction could greatly improve the quality of neural recording in a closed-loop system.

## Conclusion

In this article, the circuit structures and the latest technologies of neural recording and stimulation circuits are summarized. The key design directions of a closed-loop neural prosthesis chip and advances of neural recording and stimulation integrated circuits are introduced. Due to the different characteristics of neural recording and neural stimulation, we discuss the important parameters in the design process. The various latest technologies mentioned and an analysis of the future trend in this article could help the designers meet their performance requirements in future biomedical device development.

## Author Contributions

XL and TC: analysis of the electronic integrated circuits. WM, JL, and HY: parameters analysis and writing and revising of the manuscript. All authors contributed to the article and approved the submitted version.

## Conflict of Interest

The authors declare that the research was conducted in the absence of any commercial or financial relationships that could be construed as a potential conflict of interest.

## Publisher’s Note

All claims expressed in this article are solely those of the authors and do not necessarily represent those of their affiliated organizations, or those of the publisher, the editors and the reviewers. Any product that may be evaluated in this article, or claim that may be made by its manufacturer, is not guaranteed or endorsed by the publisher.
